# The combination of RPA-CRISPR/Cas12a and *Leptospira* IgM RDT enhances the early detection of leptospirosis

**DOI:** 10.1371/journal.pntd.0011596

**Published:** 2023-08-25

**Authors:** Sirawit Jirawannaporn, Umaporn Limothai, Sasipha Tachaboon, Janejira Dinhuzen, Patcharakorn Kiatamornrak, Watchadaporn Chaisuriyong, Nattachai Srisawat

**Affiliations:** 1 Excellence Center for Critical Care Nephrology, King Chulalongkorn Memorial Hospital, Bangkok, Thailand; 2 Faculty of Medicine, Center of Excellence in Critical Care Nephrology, Chulalongkorn University, Bangkok, Thailand; 3 Tropical Medicine Cluster, Chulalongkorn University, Bangkok, Thailand; 4 Faculty of Medicine, Division of Nephrology, Department of Medicine, Chulalongkorn University, King Chulalongkorn, Memorial Hospital, Bangkok, Thailand; 5 Academy of Science, Royal Society of Thailand, Bangkok, Thailand; Faculty of Medicine and Health Sciences, Universiti Putra Malaysia, MALAYSIA

## Abstract

**Background:**

Lack of available sensitive point-of-care testing is one of the primary obstacles to the rapid diagnosis of leptospirosis. The purpose of this study was to test the performance of two point-of-care tests, a clustered regularly interspaced short palindromic repeat (CRISPR)/CRISPR-associated protein 12a (CRISPR/Cas12a) fluorescence-based diagnostic assay (FBDA), a *Leptospira* immunoglobulin M (IgM) rapid diagnostic test (RDT), and the two tests combined.

**Methodology/Principal findings:**

For the diagnosis of 171 clinical samples, a recombinase polymerase amplification (RPA)-CRISPR/Cas12a FBDA for whole blood and *Leptospira* IgM RDT (Medical Science Public Health, Thailand) for serum were used. The confirmed cases were determined by using any positive qPCR, microscopic agglutination test (MAT), and culture results. Diagnostic accuracy was assessed on the first day of enrollment and stratified by the day after symptom onset. The overall sensitivity of the *Leptospira* IgM RDT and RPA-CRISPR/Cas12a FBDA was 55.66% and 60.38%, respectively. When the two tests were combined, the sensitivity rose to 84.91%. The specificity of each test was 63.08% and 100%, respectively, and 63.08% when combined. The sensitivity of the *Leptospira* IgM RDT rose on days 4–6 after the onset of fever, while the RPA-CRISPR/Cas12a FBDA continued to decrease. When the two tests were combined, the sensitivity was over 80% at different days post-onset of fever.

**Conclusions/Significance:**

The combination of *Leptospira* IgM RDT and RPA-CRISPR/Cas12 FBDA exhibited significant sensitivity for the detection of leptospires at various days after the onset of fever, thereby reducing the likelihood of misdiagnosis. The combination of these assays may be suitable for early leptospirosis screening in situations with limited resources.

## Author summary

Lack of available sensitive point-of-care testing is one of the primary obstacles to the rapid diagnosis of leptospirosis. This study aimed to test the performance of a CRISPR/Cas12a FBDA, a *Leptospira* IgM RDT, and a combined test. Diagnostic accuracy was assessed on the first day of enrollment and stratified by the day after symptom onset. The combined assays exhibited significant sensitivity for the detection of leptospires at over 80% on various days after the onset of fever.

## Introduction

Leptospirosis is a zoonotic disease caused by the spirochete *Leptospira* spp. With a large high impact on global health, the disease accounts for over a million cases and 58,900 deaths annually [1–3]. Leptospirosis is difficult to diagnose since its clinical signs and symptoms are similar to those of several other infectious diseases including dengue fever, sepsis, and malaria [4–7].

One of the greatest obstacles to reduce the burden of leptospirosis is the lack of sensitive diagnostic instruments, particularly point-of-care tests. Currently, the three standard methods recommended by the World Health Organization (WHO) are the microscopic agglutination test (MAT), blood culture, and polymerase chain reaction (PCR) [8]. The PCR is a nucleic acid detection method that is faster, more accurate, and commonly employed as a major diagnostic tool. However, PCR equipment is costly and is not available in every hospital, particularly in rural locations [5,9–11]. For antibody detection, the WHO also recommends the enzyme-linked immunosorbent test (ELISA). However, antibody levels during the first week following infection are frequently low thereby reducing the effectiveness of ELISA [5]. In addition, the majority of leptospirosis cases are admitted to rural hospitals that lack adequate laboratory equipment to carry out these tests. Therefore, there remains an urgent need for new diagnostic tests for leptospirosis [12].

In resource limited settings, testing at the point of care is ideal. While *Leptospira* immunoglobulin M (IgM) rapid diagnostic tests (RDTs) are currently commercially available, it, suffers from limited sensitivity and may be inefficient for early screening of acute leptospirosis, especially during the first week after symptom onset [13]. Recently, we developed an RPA-CRISPR/Cas12a-fluorescent-based detection assay (FBDA) targeting the *lipL32* gene and then demonstrated its potential utility in leptospirosis screening with 85.2% sensitivity and 100% specificity compared to qPCR [14]. The RPA-CRISPR/Cas12a FBDA can detect pathogenic *Leptospira* spp. peaking at 4–6 days after the onset of fever. However, sensitivity decreased over time, particularly on day ≥7 after fever onset [15]. The primary objective of this study was to test the performance of a CRISPR/Cas12a FBDA, a *Leptospira* IgM RDT, and the combined test in order to determine the most effective point-of-care test for diagnosis of leptospirosis.

## Materials and methods

### Ethics statement

The Chulalongkorn University Faculty of Medicine’s Institutional Review Board in Bangkok, Thailand, approved the study process (COA No.0342/2022). All participants provided written informed consent, which included usage of the remaining sample for additional research. This study adhered to international requirements for human research protection established by the Declaration of Helsinki, The Belmont Report, CIOMS Guidelines, and the International Conference on Harmonization in Good Clinical Practice.

### Sample size estimation

The total minimum sample size of 177 (106 positive samples and 71 negative samples) was calculated for 7.5% margin of error (*d*) for sensitivity estimation and 12.5% margin of error (*d*) for specificity estimation at 95% confidence level (α = 0.05), Z is the corresponding confidence level statistic, expecting the sensitivity and specificity (*p*) were of about 85% and 63% respectively [16].


$$n=\frac{Z_{1-\frac{a}{2}}^{2}p(1-p)}{d^{2}}$$

n = number of positive samples for sensitivity

n = number of negative samples for specificity

### Patients and study design

From two cohorts, a total of 171 blood and serum samples from individuals with known leptospirosis status (infected or uninfected) were obtained at the first day of enrollment, and 7 days apart for pair serum MAT testing. 110 samples were taken from previous research that were performed at 15 hospitals in the Thai region of Sisaket between December 2015 and November 2016 [17]. From January 2019 to January 2022, 61 samples were gathered from 5 hospitals in the province of Nakhon Si Thammarat. We defined first day of enrollment as the first day of leptospirosis suspicion. The inclusion criteria were that all subjects must (i) be older than 18 years old and admitted to a participating hospital; (ii) have presented with clinical suspicion of leptospirosis, high fever (body temperature higher than 38°C), severe myalgia; and (iii) have a history of exposure to reservoir animals. The exclusion criteria were patients who suffered from other known infectious diseases. The Blood samples were taken on the first day of enrollment and the seventh day after enrollment. Blood samples were maintained at a temperature of 4°C while being transported from the site of blood collection to our laboratory, and were then refrigerated at -80°C until analysis. Total DNA was extracted from 200 μL of whole blood samples for clinical sample validation using the High Pure PCR Template Preparation Kit (Roche, USA) according to the manufacturer’s instructions. A NanoDrop 2000 was used to determine the concentration and quality of the extracted DNA (Thermo Scientific, USA). The serum was kept at -80°C until the *Leptospira* IgM RDT was performed. Samples from the first day of enrollment were selected and used as a blind test.

### Leptospirosis case definition

We confirmed leptospirosis using three WHO-recommended methods: MAT, direct blood culture, and PCR in blood [8]. In this study, we use qPCR to enhance the effectiveness of diagnostic test as described in [18].

If any of the three tests were positive, patients with suspected leptospirosis were categorized as confirmed cases. MAT tests were conducted on 24 serovars of *Leptospira interrogans*. Australis, Autumnalis, Ballum, Bataviae, Canicola, Cellidoni, Cynopteri, Djasiman, Grippotyphosa, Hebdonadis, Icterohaemorrhagiae, Javanica, Louisiana, Manhao, Mini, Panama, Pomona, Pyrogenes, Ranarum, Sarmin, Sejroe, Shermani, Tarasovi, and Semaranga. Positive MAT was defined as a single serum titer of 1:800 or a fourfold rise in pair serum titers (7 days apart) based on CDC definition 2013 [19]. For the direct culture of leptospirosis, 1 mL of whole fresh blood was added to 4 mL of Ellinghausen, McCullough, Johnson and Harris (EMJH) medium with a syringe, and the mixture was incubated at 30°C as described in [20] with total volume of 5 mL from 10 mL for cost-effectiveness. Leptospires were detected by direct observation with dark-field microscopy every two weeks up to 18 weeks. The qPCR targeting the *lipL32* gene was conducted as described before [18] with slight modifications. Using the forward primer (5′- AAG CAT TAC CGC TTG TGG TG -3′), reverse primer (5′- GAA CTC CCA TTT CAG CGA TT -3′), and Taqman probe (5′-FAM- AA AGC CAG GAC AAG CGC CG-BHQ1-3′), 242 base pair products were amplified and identified. In a final volume of 20 μL, the qPCR mixture contained 5 μL of extracted DNA, 10 μL of SsoAdvanced Universal Probe Supermix (Bio-Rad Laboratories, USA), 1 μL of each primer (10 μM), 0.4 μL of Taqman probe (10 μM), and 2.6 μL of nuclease-free water. As a negative control, a no template control (NTC) containing all of the mentioned reagents was utilized. The StepOnePlus qPCR System was utilized for amplification and fluorescence detection (Applied Biosystems, USA). The amplification procedure consisted of 10 minutes at 95°C, followed by 45 cycles of 15 seconds at 95°C, and one minute at 60°C. A result was determined to be negative if the threshold cycle (Ct) value exceeded 40 cycles.

### The RPA-CRISPR/Cas12a FBDA

The RPA-CRISPR/Cas12a FBDA targeting the *lipL32* gene was performed as previously reported with a few minor changes [14]. RPA was done using the same primer set as the qPCR utilizing the TwistAmp Basic Kit (TwistDx, United Kingdom). Briefly, rehydrated lyophilized RPA was combined with 480 nM of each primer in rehydration buffer. Then, 18 mM of magnesium acetate (final concentration) and 5 μL of extracted DNA were added to the reaction mixture with 25 μL total volume. The *lipL32* gene was amplified by incubation at 39°C for 40 minutes, followed by inactivation at 75°C for 5 minutes. We then performed CRISPR/Cas12a FBDA detection using crRNA. The CRISPR/Cas12a reaction contained 30 nM of crRNA (5’-UAAUUUCUACUAAGUAGAUUUCUGAGCGAGGACACAAUC-3’), 330 nM of EnGen Lba Cas12a (Cpf1) (New England Biolabs, USA), 600 nM of fluorescent probe (5’-FAM-TTATTATT-BHQ1-3’), and 1X of NEBuffer 2.0 (New England Biolabs, USA). The total volume was 15μL. Twenty minutes were spent incubating the CRISPR/Cas12a reaction at 39° C. The fluorescent signal was then detected using a BluePAD Dual LED Blue/White Light Transilluminator (BIO-HELIX, Taiwan) with a 470 nm wavelength. Each qualitative test was seen by three qualified laboratory technicians who were trained to classify the result as "positive" or "negative." The tests were deemed positive if at least two of the three technicians interpreted the results as positive. The outcome was unknown to the technicians.

### *Leptospira* IgM rapid diagnostic testing

On the first day of enrollment, the *Leptospira* IgM RDT (Department of Medical Sciences, Ministry of Public Health, Thailand) was chosen to analyze the sera of 171 individuals. The *Leptospira* IgM RDT kit was used in accordance with the manufacturer’s instructions to detect *Leptospira* IgM antibodies. The serum sample was thawed at room temperature before being transferred, without air bubbles, to the sample well in a volume of 10 μL. The five drops of assay diluent were then dropped into the diluent well. After 15 minutes, three qualified technicians interpreted the results. If at least two of the three technicians interpreted the data as affirmative, the tests were deemed positive.

### Statistical analysis

Continuous variables are displayed as the mean ± one standard deviation (SD) if the distribution is normal, and as the median and interquartile range (IQR) if the distribution is not normal. The Mann-Whitney test was used to analyze the differences between days after onset of symptoms for each test using the GraphPad Prism 9.5.1 (GraphPad Software Inc., California, USA). The performance of the RPA-CRISPR/Cas12a targeting the *lipL32* gene detection system and *Leptospira* IgM RDT was evaluated by calculating the sensitivity, specificity, accuracy, and positive and negative predictive values at 95% confidential interval and then compared to the three standard methods (qPCR, MAT, and direct blood culture) of the same samples using MedCalc Software Ltd. Diagnostic test evaluation calculator. https://www.medcalc.org/calc/diagnostic_test.php (Version 20.118; accessed November 24, 2022). The McNemar test was used to analyze the differences in performance of each test overall and by day after onset of symptoms using the STATA software version 17 (StataCorp LLC, Texas, U.S.) [21].

## Results

### Study population

**Fig 1:**
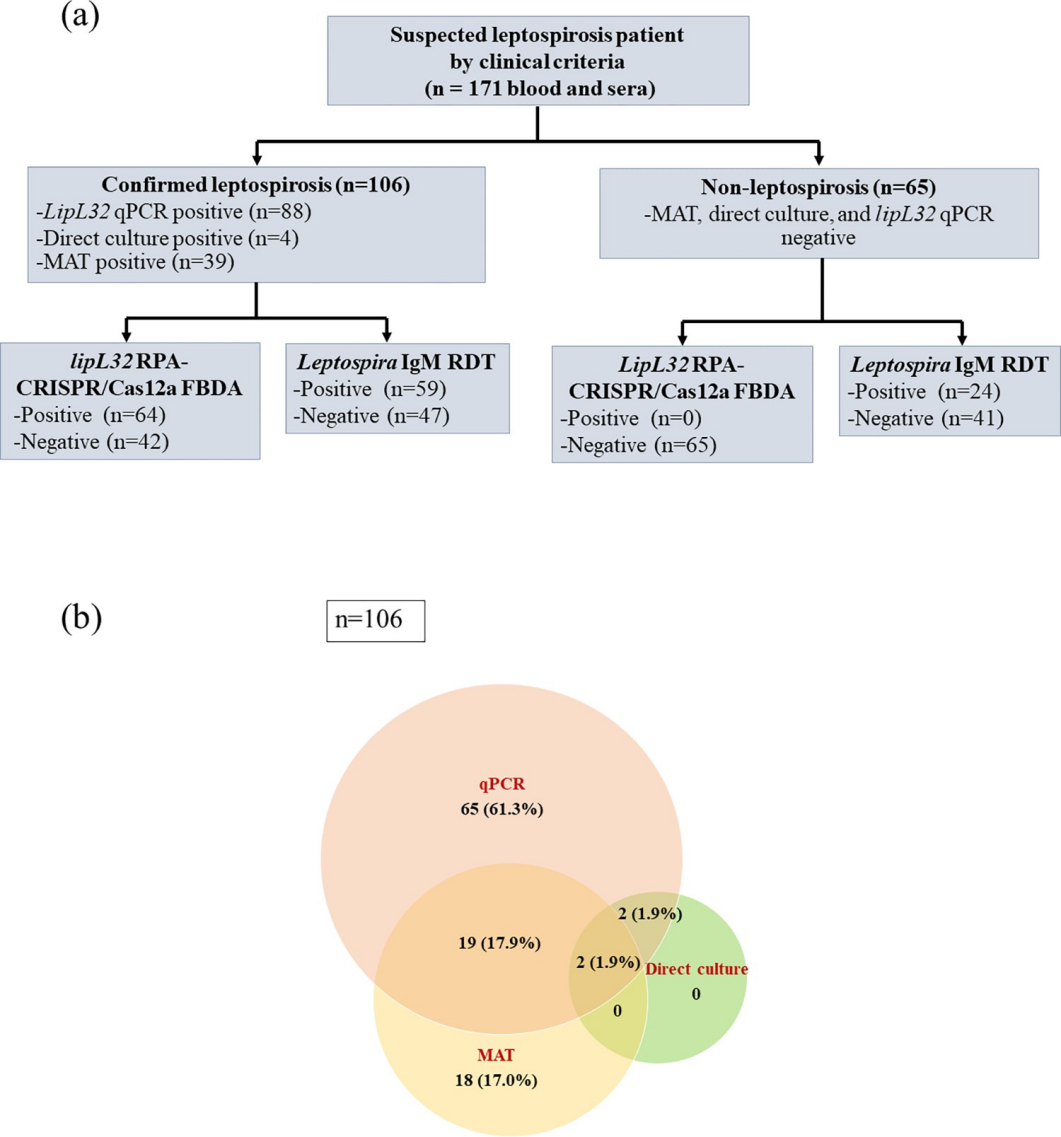
(a) The sampling approach used in this study, the diagnostic tests performed, and the number of positive and negative samples obtained for each test. (b) The Venn diagram created by Microsoft PowerPoint 2021(US) represented the number of positive cases diagnosed by each standard method.

**Fig 2:**
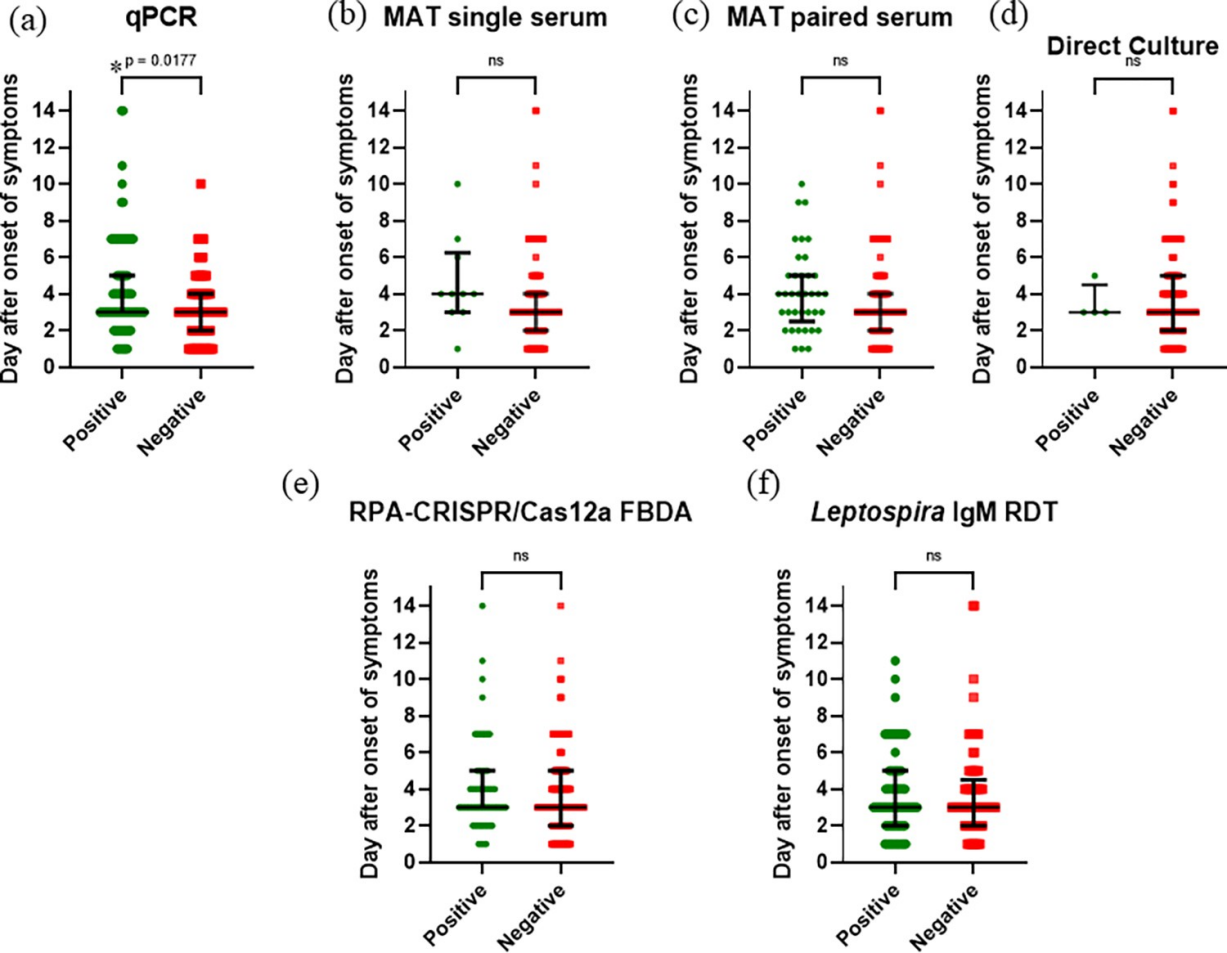
The median and interquartile range (IQR) of days since symptom onset for each diagnostic test were calculated using GraphPad Prism 9.5.1 (GraphPad Software Inc., California, USA). *P*-values ≤ 0.05 are considered statistically significant. (a) qPCR (b) MAT (day 0) (c) MAT (day 7) (d) direct culture (e) RPA-CRISPR/Cas12a FBDA (f) *Leptospira* IgM RDT.

Out of 171 patients tested for leptospirosis, 106 (62%) were confirmed cases of leptospirosis and 65 (38%) were non-leptospirosis cases. Among 106 confirmed leptospirosis patients, 71 (67%) were qPCR positive, 4 (3.7%) were culture positive, and 38 (35.8%) were MAT positive (**Fig 1A and 1B**). **Fig 2** shows the comparison of days after onset of symptoms for each assay. The qPCR revealed statistically significant differences between positive and negative test result (*p*<0.05). There was no difference for other diagnostic tests on the days following the onset of symptoms.

### Diagnostic performance of *Leptospira* IgM RDT, RPA-CRISPR/Cas12a FBDA and both tests combined

**Fig 3:**
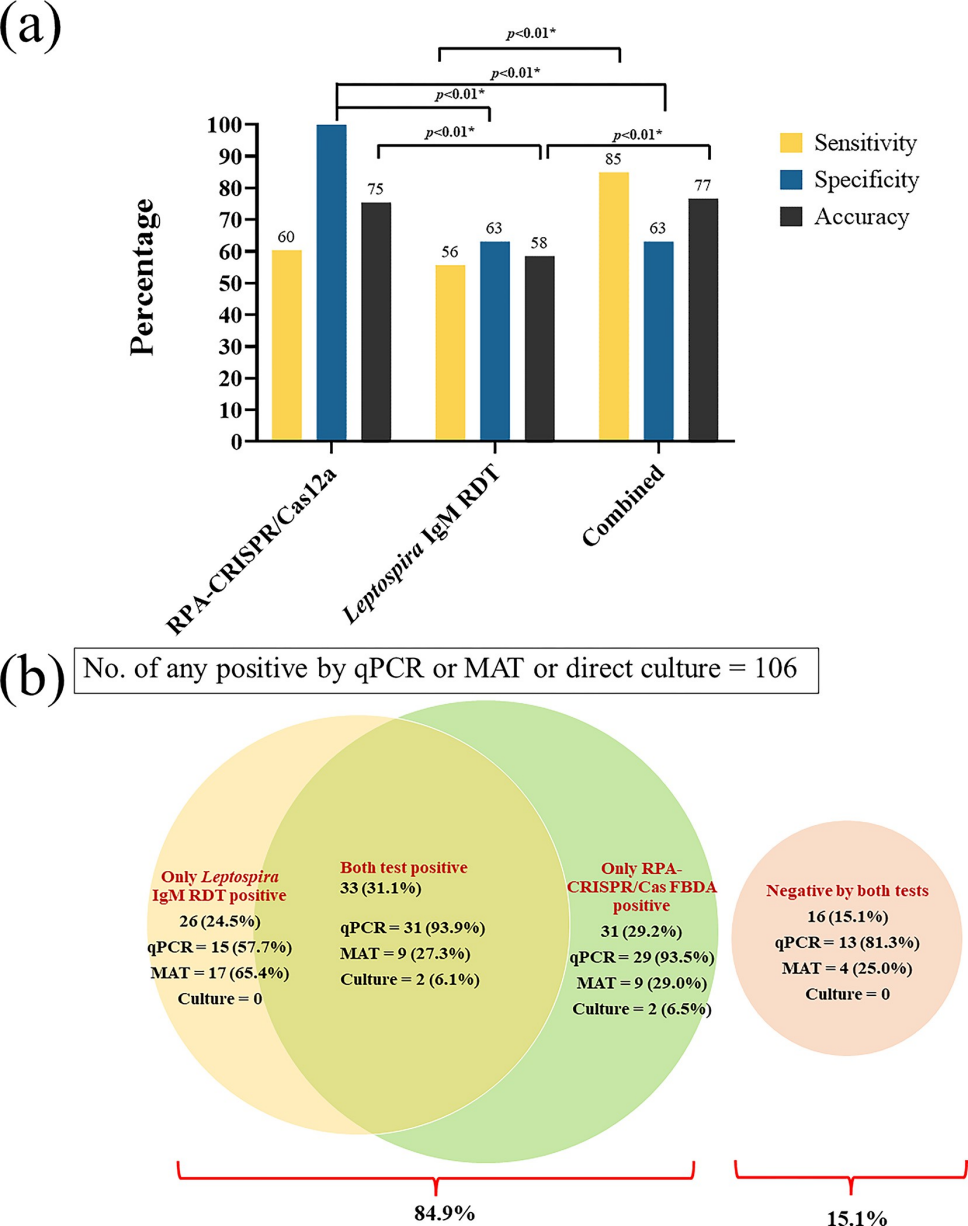
Comparison of the overall performance of RPA-CRISPR/Cas12a FBDA, *Leptospira* IgM RDT, and both tests combined. (a) Comparison of each individual test’s specificity, sensitivity, and accuracy to the combined test. *P*-values ≤ 0.05 are considered statistically significant. Figs were made using GraphPad Prism 9 (GraphPad Software Inc., California, USA). (b) The Venn diagram created by Microsoft PowerPoint 2021(US) represented the number of cases diagnosed by each technique when compared to standard methods. The distribution of standard methods in each section was represented by a positive number and a percentage distribution.

The RPA-CRISPR/Cas12a FBDA and *Leptospira* IgM RDT combination has the highest overall sensitivity at 85%. RPA-CRISPR/Cas12a FBDA alone demonstrated the highest specificity at 100%. RPA-CRISPR/Cas12a FBDA alone and combined test demonstrated accuracy of 75% and 77%, respectively (**Fig 3A and Table 1**). The qPCR median of CT is 32.23 (27.81, 34.88) from 88 positive samples. For MAT single serum, the minimum was <1:50 and maximum was 1:3200. For MAT paired serum, the minimum was 1:200, the maximum was 1:12,800. In addition, the Venn diagram revealed that positive result for each test has either intersected or distinct population. When both tests were combined, only 16 cases were missed (15.1%) (**Fig 3B**).

**Table 1:** Performance of RPA-CRISPR/Cas12a FBDA, *Leptospira* IgM RDT and both tests combined.

Parameter	CRISPR/Cas12a FBDA	*Leptospira* IgM RDT	Combined
Total samples	171	171	171
True positive (TP)	64	59	90
True negative (TN)	65	41	41
False positive (FP)	0	24	24
False negative (FN)	42	47	16
Sensitivity	60.38%	55.66%	84.91%
Specificity	100.00%	63.08%	63.08%
Positive Predictive Value (PPV)	100.00%	71.08%	78.95%
Negative Predictive Value (NPV)	60.75%	46.59%	71.93%
Accuracy	75.44%	58.48%	76.61%

### Diagnostic performance at different day post-onset of fever

**Fig 4:**
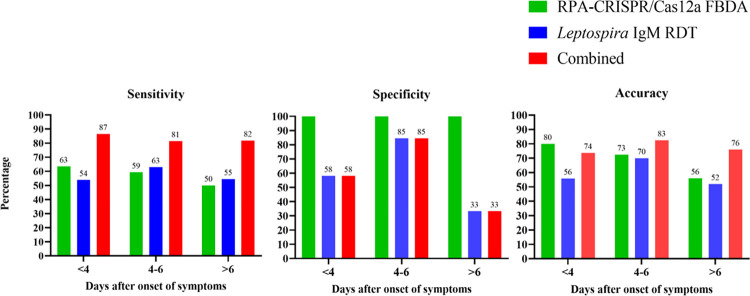
Comparison of the overall performance of RPA-CRISPR/Cas12a FBDA, *Leptospira* IgM RDT, and both tests at various days after symptom onset. Figs were made using GraphPad Prism 9 (GraphPad Software Inc., California, USA).

**Fig 5:**
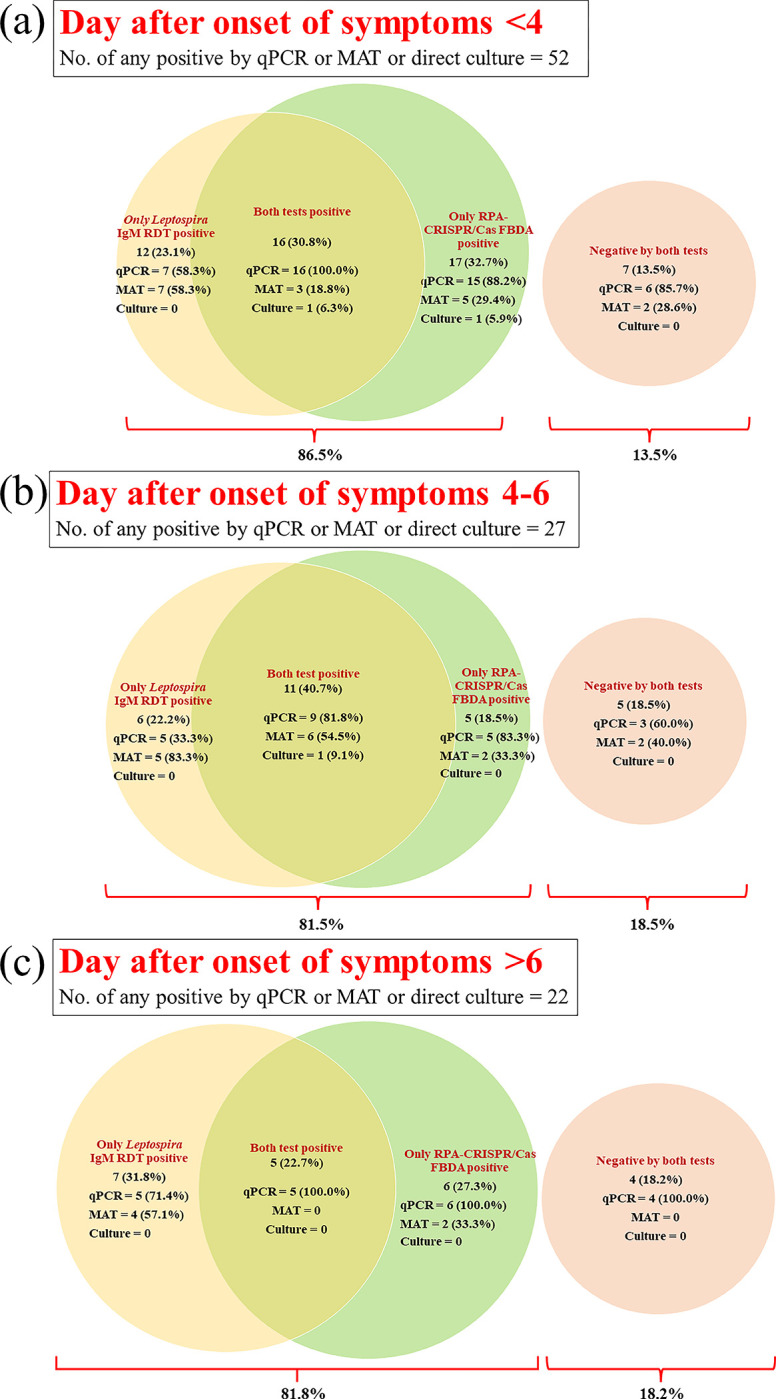
The Venn diagram shows the number of cases diagnosed by each technique in comparison to standard methods at various days after symptom onset. (a) Day <3 (b) Day 4–6 (c) Day >6. A positive number and a percentage distribution were used to depict the distribution of standard methods in each section calculated by number of positive by each standard test/number of positive by point-of-care tests*100.

**Fig 6:**
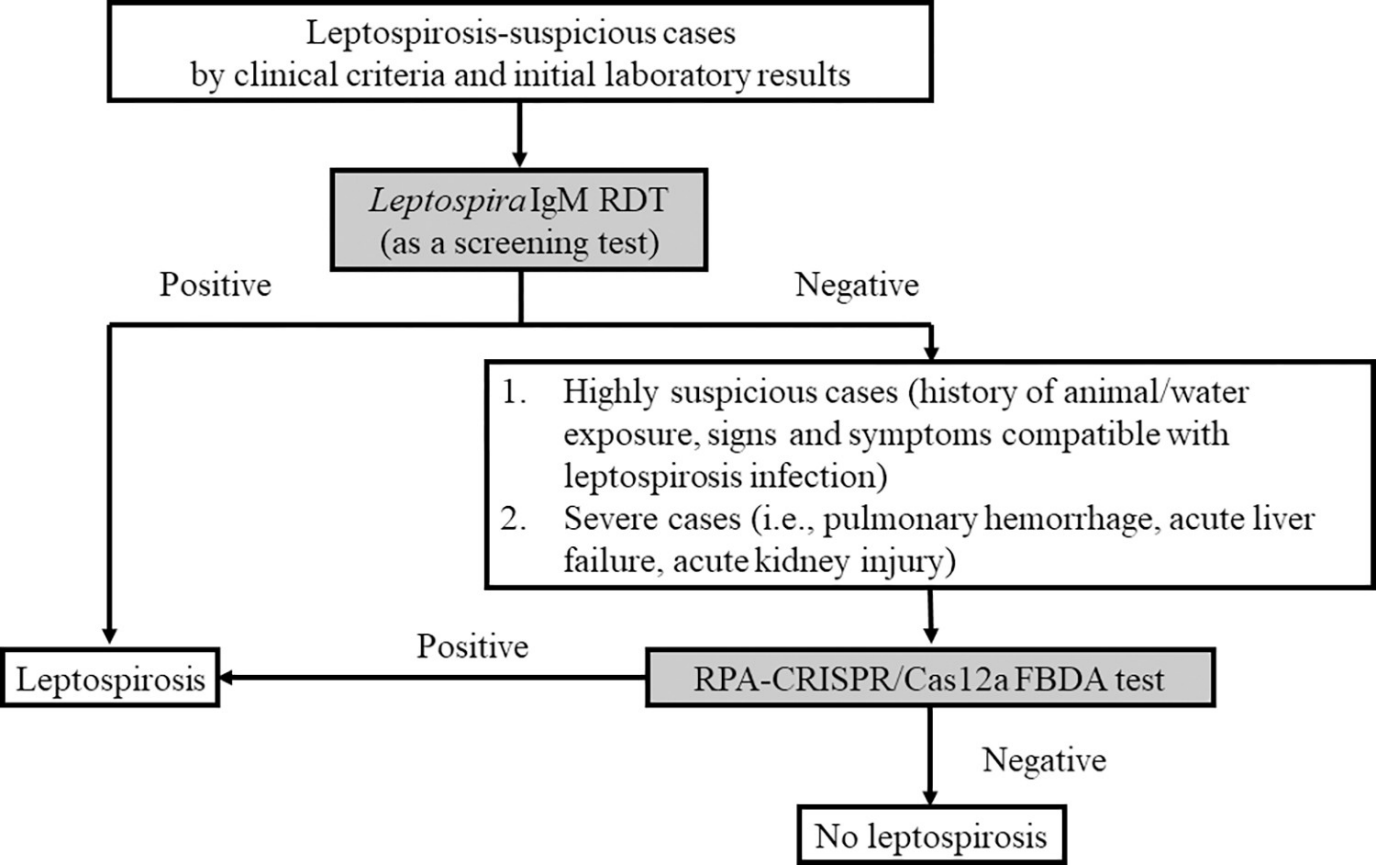
Proposed decision algorithm to screen for leptospirosis by using a *Leptospira* IgM RDT and an RPA-CRISPR/Cas12a FBDA assay.

To examine trend of the test performance categorized by days after symptom onset, we divided the patients into three subgroups depending on the day after symptoms started: day <4 (n = 95), day 4–6 (n = 40), and day >6 (n = 25). Eleven individuals lacked data on the day after the beginning of fever. The results showed no difference in sensitivity between the RPA-CRISPR/Ca12a FBDA and the *Leptospira* IgM RDT. When both tests were combined, however, sensitivity increased by more than 80 percent while specificity decreased. For accuracy, RPA-CRISPR/Cas12a FBDA had the highest accuracy on the first three days, whereas the combined test had the highest accuracy on day 4, as shown in **Fig 4**. Furthermore, the Venn diagram demonstrates that when both tests are combined, the missing cases are minimized to 13.5–18.5%. (**Fig 5A and 5C**). Fig 6 demonstrates the decision-making process for screening for leptospirosis using a *Leptospira* IgM RDT and an RPA-CRISPR/Ca12a FBDA assay based on our findings.

## Discussion

Point-of-care diagnostic tests are critical for detecting leptospirosis in limited resource settings, where the majority of cases in Thailand have occurred. This cross-sectional study demonstrated the efficiency of combining two point-of-care approaches for leptospirosis identification on the first day of enrollment.

Our study revealed that RPA-CRISPR/Cas12a FBDA and *Leptospira* IgM RDT may have comparable sensitivity, but RPA-CRISPR/Cas12a FBDA has significantly higher specificity and accuracy. The combined tests enhanced the overall sensitivity by over 80% while decreasing the specificity from 100% to 63%. However, the Venn diagram suggested that the positive populations for each test either overlapped or were distinct, and by combining both tests, they covered true positives by 84.9% (**Fig 3B**). In addition, the overall PPV and NPV in **Table 1** for combined methods were 78.96% and 71.93%, respectively, representing a balance between false negatives lost and false positives gained when compared to each individual method. Therefore, the combined test is the best option for detecting as many true positives as possible. The use of a single method, such as RPA-CRISPR/Cas12a FBDA or *Leptospira* IgM RDT, may miss cases and result in a misdiagnosis.

The *Leptospira* IgM RDT (Department of Medical Sciences, Ministry of Public Health, Thailand) has a 15-minute turn-around time, and the cost per test is 2.60 USD. However, for RPA-CRISPR/Cas12a FBDA has a turn-around time of 85 minutes, as described in our previous study [14], and the cost per test is difficult to determine due to small-scale research experiments, not mass-scale production. However, for our lab-scale test, the cost is around 14.45 USD. *Leptospira* IgM RDT is a good option for leptospirosis screening due to its cost-effectiveness and quick turnaround time.

According to our study, utilizing both tests would increase the cost. Therefore, we proposed two stepwise approaches for screening human leptospirosis. First, we suggest using the *Leptospira* IgM RDT as the first screening test. If the test is negative but the clinicians are still highly suspicious of leptospirosis including patients with severe clinical manifestation/organ failure, we may consider using RPA-CRISPR/Ca12a FBDA as the second screening test to prompt leptospirosis diagnosis. With this approach, we can increase leptospirosis detection rate from 56% to 85% (**Fig 6**).

This study evaluated the RPA-CRISPR/Cas12a FBDA and the *Leptospira* IgM RDT in comparison to three WHO-recommended standard methods: *lipL32* qPCR, MAT, and direct culture. **Fig 1B** demonstrates that when *lipL32* qPCR is negative, MAT has a 17% positive rate. When compared to only *lipL32* qPCR, the sensitivity of RPA-CRISPR/Cas12a FBDA was increased from 60.38% to 72.2%. Of the 16 cases missed by RPA-CRISPR/Cas12a LFDA and *lipL32* RDT tests, the qPCR was responsible for 13 cases (81.3%) with the Ct ranging from 34.36–38.42, while MAT was responsible for 4 cases (25%). The qPCR is a standard sensitive diagnostic technique. In our settings, the limit of detection (LOD) in pure *Leptospira* spp. culture for qPCR was reached at 10^1^ cells/mL, whereas RPA-CRISPR/Cas12 FBDA has 10^2^ cells/mL. In addition, we compared the RPA-CRISPR/Cas12a FBDA positive group and negative group using qPCR Ct (**S1 Fig**). The results revealed statistically significant differences between the two groups (*p*>0.0001). The median qPCR Ct of the negative CRISPR/Cas12a FBDA group is 35.17 (34.42, 36.97), while the median qPCR Ct of the positive group is 30.11 (25.85, 33.07). Consequently, the missing cases and decreased sensitivity compared to our previous study were due to the difference in detection limit [14].

In this study, we increased the total volume of RPA reaction from 6.25 μL to 25 μL, allowing us to increase the volume of the template from 1μL to 5μL in order to increase the test’s sensitivity. For MAT test, a small number of serovars may produce false-negative MAT results [11]. For an effective MAT test, further research is necessary to identify the pathogenic *Leptospira* spp. serovar in Thailand. Additionally, qPCR demonstrated a significant difference between the positive and negative groups (**Fig 2A**). The median values were the same (3 days after onset of symptoms), but the overall distribution was not the same, and the interquartile range (IQR) for these two groups was distinct: 3 to 5 days for the qPCR-positive group and 2 to 4 days for the qPCR-negative group.

When test performance was categorized by day after the onset of symptoms, the results were similar to overall performance. The sensitivity of RPA-CRISPR/Cas12a FBDA peaked on day < 4 after the onset of fever and decreased with time whereas sensitivity of *Leptospira* IgM RDTs increased on day 4–6 and declined on day > 6, similar to previous findings [13,14,22].

Leptospires are normally present in the blood during the first week and are eliminated by the host-immunity while IgM and IgG antibody levels begin to rise and reach their peak during the second week of illness [11]. The anti-*Leptospira* IgM and IgG can stay in the blood for months or even years [5,9–11]. However, our results demonstrated early detection of IgM within three days after symptom onset. Due to the reason that the samples were collected in a high-risk region in Thailand [12,23], early detection of *Leptospira* IgM may indicate a previous infection with pre-existing IgM [11] or resulted from a rapid immune response to secondary infection, as other infectious diseases demonstrated [24,25]. Due to the small sample size, the specificity of the *Leptospira* IgM RDT on day > 6 after the onset of symptoms was low (true negative = 3). Therefore, the results should be interpreted cautiously. In addition, we have found that *Leptospira* spp. may decrease in blood after day 7, however, due to the low LOD of qPCR it was possible to get positive result as shown in our study.

There was a publication that successfully developed the *Leptospira* antigen test utilizing *lipL32* from Thailand [26]. However, this study only evaluated the limit of detection using *Leptospira interrogans* serovar Bratislava, and it was unclear whether the limit of detection for other serovars would be different. Further studies may be needed to validate the effectiveness of this immunochromatographic assay for *Leptospira* detection in different serovars.

*Leptospira* IgM RDT and RPA-CRISPR/Cas12a FBDA are methods that need minimal equipment, making them appropriate for field testing or in hospitals in remote areas. Using them together improved sensitivity while reducing the number of missed leptospirosis cases.

We identified four strengths of the study. First, our study was conducted utilizing blinded evaluations by three separate observers to reduce bias. Second, we confirmed leptospirosis patients using the three standard criteria, which is crucial for determining test accuracy. Third, our results demonstrated that the sensitivity of the two point-of-care tests did not differ overall or by day after symptom onset. Therefore, we used a Venn diagram to show the positive results of either RPA-CRISPR/Ca12a FBDA or *Leptospira* IgM RDT could be found in the same populations or distinct **(Fig 3B).**

There are also several limitations in this study. First, patients arrived at the hospital at varying time periods after symptom onset. As a result, we were unable to assess all patients on the first day of fever. Second, since the majority of patients arrived at the hospital shortly after symptom onset (first week), only a small number of patients (n = 27) on day 4–6 and (n = 22) on day > 6. Therefore, we did not analyze and compare the performance of the tests stratified by day after symptom onset and the results should be interpreted with caution. Third, our study was designed for leptospirosis detection on the first day of enrollment and not to follow patients every day after the onset of symptoms. Consequently, our results revealed only the general performance trend of the test based on the information we possess. Third, since clinical samples were gathered from endemic regions in Thailand, our findings may lack generalizability to the other parts of the world, especially in non-endemic areas. Finally, this is a cross-sectional study of novel RPA-CRISPR/Cas12a FBDA and *Leptospira* IgM RDT for leptospirosis detection. Currently, we are prospectively conducting a cohort study to validate the role of novel RPA-CRISPR/Cas12a FBDA in improving the rate of leptospirosis detection in the real clinical setting.

In conclusion, the combination of *Leptospira* IgM RDT and RPA-CRISPR/Cas12a FBDA greatly increased sensitivity and specificity over the conventional approach of using only one test. These findings are crucial to improving early detection of leptospirosis and administering early treatment. Both tests are affordable, portable, and rapid, making them suitable for use in the field, especially in rural hospitals with limited resources.

## Supporting information

S1 Fig The qPCR Ct comparison between the RPA-CRISPR/Cas12a FBDA positive and negative groups. The graph, median, and interquartile range (IQR) of qPCR positive and negative group were calculated using GraphPad Prism 9.5.1 (GraphPad Software Inc., California, USA). *P*-values ≤ 0.05 are considered statistically significant.
(DOCX)
