# Supplementary material for: The combination of RPA-CRISPR/Cas12a and Leptospira IgM RDT enhances the early detection of leptospirosis
Source: PLoS Negl Trop Dis. 2023 Aug 25;17(8):e0011596. doi: 10.1371/journal.pntd.0011596 (PMC10497128; doi:10.1371/journal.pntd.0011596)
Supplement: S1 Fig — The graph, median, and interquartile range (IQR) of qPCR positive and negative group were calculated using GraphPad Prism 9.5.1 (GraphPad Software Inc., California, USA). P-values ≤ 0.05 are considered statistically significant. (DOCX) [file pntd.0011596.s001.docx]

**S1 Fig.** The qPCR Ct comparison between the RPA-CRISPR/Cas12a FBDA positive and negative groups. The graph, median, and interquartile range (IQR) of qPCR positive and negative group were calculated using GraphPad Prism 9.5.1 (GraphPad Software Inc., California, USA). *P*-values ≤ 0.05 are considered statistically significant.
